# Dandelion polysaccharide treatment protects against dextran sodium sulfate‐induced colitis by suppressing NF‐κB/NLRP3 inflammasome‐mediated inflammation and activating Nrf2 in mouse colon

**DOI:** 10.1002/fsn3.3653

**Published:** 2023-09-19

**Authors:** Shuo Wang, Ping Wu, Zongqiang Fan, Xingrui He, Jinqian Liu, Ming Li, Fang Chen

**Affiliations:** ^1^ School of Pharmaceutical Sciences Liaocheng University Liaocheng Shandong China; ^2^ School of Pharmacy Hangzhou Normal University Hangzhou Zhejiang China; ^3^ Shandong Academy of Occupational Health and Occupational Medicine Shandong First Medical University & Shandong Academy of Medical Sciences Jinan Shandong China

**Keywords:** dandelion polysaccharide, NF‐κB, NLRP3, Nrf2/HO‐1 pathway, ulcerative colitis

## Abstract

The treatment of ulcerative colitis (UC) is still an intractable medical problem. Polysaccharides are promising candidates for the treatment of UC and have received widespread attention in recent years. The objective of this study was to explore the protective effect and underlying mechanism of dandelion polysaccharide (DP) on dextran sulfate sodium (DSS)‐induced colitis in mice. Our results showed that oral administration of DP could dramatically alleviate colonic lesions, as evidenced by reduced DAI scores, shortening of colon length, and ameliorating pathologic abnormalities in colons. Additionally, the expressions of pro‐inflammatory factors (TNF‐α, IL‐1β, and IL‐6) and the infiltration of inflammation‐regulation cells, marked by myeloperoxidase and F4/80, were also inhibited after DP treatment. Moreover, DP treatment also markedly suppressed the nuclear translocation of NF‐κB‐p65 and the activation of the NLRP3 inflammasome. Furthermore, DP also activated the Nrf2/HO‐1 pathway and reduced the oxidative stress induced by DSS. Overall, these results suggest that DP could be a promising novel therapeutic approach for the treatment of UC.

## Graphical abstract

Dandelion polysaccharides (DP) ameliorated DSS‐induced colitis in the mouse model. DP treatment suppressed oxidative stress and inflammation in colitis. DP inhibited the activation of the NF‐κB/NLRP3 pathway and upregulated the Nrf2/HO‐1 pathway to protect against colitis.

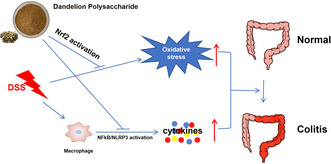

## 1 INTRODUCTION

Ulcerative colitis (UC) is one of the most prevalent inflammatory bowel diseases (IBDs), which generally cause long‐lasting inflammation ulcers in the mucosal layer with unclear pathogenesis (Pergolizzi et al., 2021). Patients with UC exhibit recurrent clinical symptoms of abdominal pain, diarrhea, and body weight loss. The current therapeutic agents for UC can only relieve disease symptoms, and with long‐term use, some patients show no response or become refractory (Armuzzi & Liguori, 2021). Therefore, it is urgent to develop new therapeutic strategies with fewer side effects and stronger efficacy.

The exact etiologies of UC remain unclear and relate to multiple factors, such as genetic susceptibility, mucosal immune dysregulation, enteric bacteria, and environmental factors (Kobayashi et al., 2020). In particular, plenty of previous studies have identified that the imbalance between pro‐ and anti‐inflammatory cytokines is one of the key pathogenic drivers of this disease (Friedrich et al., 2019). The production of pro‐/anti‐inflammatory cytokines is regulated by multiple signal pathways, among which the nuclear factor kappa B (NF‐κB) pathway and the Nod‐like receptor protein 3 (NLRP3) inflammasome have received much attention recently (Atreya et al., 2008; Zhen & Zhang, 2019). The expression and activation of both NF‐κB and NLRP3 inflammasomes are strongly induced in the inflamed gut of UC patients (Rogler et al., 1998; Zaki et al., 2011). Besides, previous studies have demonstrated that oxidative stress conspires with cytokines in time and space during the inflammatory process of UC (Moura et al., 2015). Oxygen free radicals could aggravate the release of inflammatory mediators and directly destroy the intestinal mechanical barrier (Xie et al., 2022; Zhao et al., 2020). Therefore, therapeutic interventions that target oxidative stress and inflammation appear to be the ideal strategy for handling this disease.

Polysaccharides purified from plants, fungi, and seaweed have received increasing attention due to their biological activities in several models of diseases (Li et al., 2022; Yu et al., 2018, 2022). Due to their nontoxic and beneficial properties, some bioactive polysaccharides have already been used in medical and practical applications (Yang & Zhang, 2009). Besides, it is reported that polysaccharides extracted from natural resources, such as astragalus, dendrobium officinale, and aloe, also have striking therapeutic effects on UC, indicating that polysaccharides are promising candidates for UC treatment (Liang et al., 2018; Lv et al., 2017). Dandelion is a perennial herbaceous herb with edible and medicinal values and is widely distributed in temperate regions of the northern hemisphere. Dandelion and its formulations have the potential to treat several diseases, such as diabetes mellitus, hepatitis, cancer, and bacterial and viral infections (Du et al., 2022; Yu et al., 2022; Zhao et al., 2022). These pharmacology applications are related to its anti‐tumor, anti‐inflammatory, antioxidative, and antimicrobial activities. Some progress has been made for its anti‐inflammatory pathways. It had been verified that dandelion could modulate the NF‐κB pathway and inhibit NLRP3 inflammasome activation (Yang et al., 2021). In addition, a recent study also demonstrated that dandelion root extract exerted protective effects on DSS‐induced colitis by repressing NF‐κB and inducing heme oxygenase‐1 (HO‐1) (Han et al., 2017). Dandelion polysaccharide (DP) is one of the main bioactive compounds of dandelion, and is mainly composed of glucose, galactose, arabinose, arabinose rhamnose, and glucuronic acid (Cai et al., 2017; Chen et al., 2016). Several studies have shown that DP exhibits the same bioactivities as dandelion, such as anti‐inflammatory, antioxidant, immunomodulation, antibacterial, and anti‐cancer (Cmp et al., 2014; Ding & Wen, 2018). All this evidence implied that DP might function as a potential candidate for the treatment of UC by regulating the inflammatory response and oxidative stress. To elucidate this hypothesis, the current study was designed to investigate the protective effects and underlying mechanisms of DP on DSS‐induced UC in mice.

## 2 MATERIALS AND METHODS

### 2.1 Chemicals and reagents

DP (>98% purity) was obtained from Ci Yuan Biotechnology Co., Ltd., Shanxi (Xian, China). Dextran sulfate sodium salt (DSS) (MW 36,000–50,000) and paraformaldehyde were purchased from Macklin Co. Ltd. (Shanghai, China). Mouse tumor necrosis factor‐a (TNF‐α), interleukin‐1β (IL‐1β), and interleukin‐6 (IL‐6) enzyme‐linked immunosorbent assay (ELISA) kits were purchased from R&D (Minneapolis, MN, USA). Myeloperoxidase (MPO), malondialdehyde (MDA), and nitric oxide (NO) commercial assay kits were bought from JianCheng Bio Co., Ltd. (Nanjing, China). The antibodies used in this study were listed in the Table S1. Western blotting detection reagents (ECL kits) were provided by Millipore Corp. (Bedford, MA, USA). BCA™ protein assay kits were purchased from Pierce Biotechnology, Inc. (Rockford, IL, USA). All other reagents were obtained from Sigma, unless otherwise stated.

### 2.2 Animals and treatment

**FIGURE 1:**
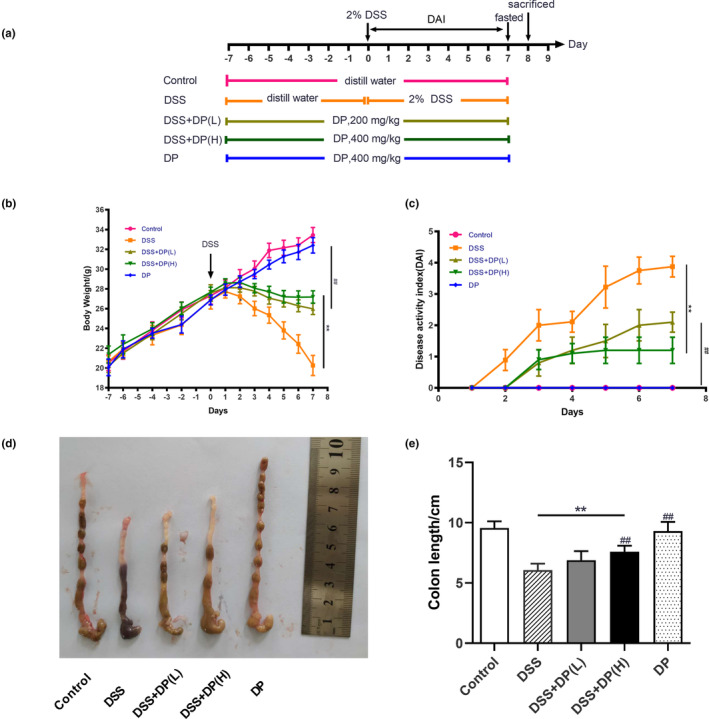
Effect of DP on the symptoms of DSS‐induced ulcerative colitis in mice. (a) Animal feeding schedule. (b) The change in body weight of mice (g), (c) the disease activity index score; (d) representative images of the colon; and (e) colon length (cm). All data are represented as the mean ± SD (*n* = 8), *p < .05, **p < .01 vs Control group; ^#^ *p* < .05, ^##^ *p* < .01 vs DSS group.

Fifty male ICR mice (18–22 g, 6–8 weeks) were purchased from Jinan Pengyue Experimental Animal Breeding Co. Ltd. (Jinan, China). All the animals were fed standard chow and housed in a temperature‐controlled room (22 ± 2°C) with a 12‐h light/dark cycle and 50–55% humidity. After acclimation for 7 days, the mice were randomly categorized into 5 groups (*n* = 8 in each group): control group, DSS group, DSS + DP(L) group (200 mg/kg/day), DSS + DP(H) group (400 mg/kg/day), and DP group (400 mg/kg/day) (Figure 1a). The doses of DP selected were based on previous reports in the literature (Ren et al., 2020). Colitis was induced with 2% DSS in drinking water for 7 days as described (Wirtz et al., 2017). DP was orally administered 7 days before the treatment of DSS until completion. The mice in the control group received an equal volume of vehicle. At the end of the experiment, the mice were sacrificed after fasting for 12 h. Blood was collected by the eyeball extract method and centrifuged to obtain serum. The colon tissue was separated, measured, and photographed. Then, the upper portion of the colons was fixed in 4% paraformaldehyde for histopathology and immunohistochemistry, while the remaining portion was quickly frozen in liquid nitrogen.

### 2.3 Assessment of disease activity index (DAI)

The body weight, stool consistency, and fecal occult blood status of mice in each group were monitored daily. The extent of colitis was evaluated by DAI score based on the average score of these three parameters according to the criteria described by Wirtz et al. (2017). The score was assigned as follows: body weight loss: none = 0, 1–5% = 1, 6–10% = 2, 11–20% = 3, >18% = 4; stool consistency: normal = 0, soft = 2, watery diarrhea = 4; and fecal blood: negative = 0, blood traces = 2, gross rectal bleeding = 4.

### 2.4 Histological analysis

Fixed colon tissues were embedded in paraffin, sectioned at 5 μm, and stained with a commercial hematoxylin and eosin (H&E) kit (Xiamen Maiwei Biotech, China). The histopathological changes of each group were examined with an Olympus BX53 microscope and scored as previously described (Sun et al., 2018). Briefly, each colon was scored considering (1) the severity of inflammation (0, none; 1, mild; 2, moderate; 3, severe); (2) the extent of inflammation (0, none; 1, mucosa; 2, mucosa and submucosa; 3, transmural); and (3) crypt damage (0, none; 1, 1/3 damaged; 2, 2/3 damaged; 3, crypt loss but surface epithelium present; 4, both crypt and surface epithelium lost). Scores were then added, resulting in a total histological score that ranged from 0 to 10.

### 2.5 Western blotting analysis

Colon tissues were weighed and lysed in RIPA lysis buffer containing protease inhibitors on ice, and then centrifuged at 12,000 *g* for 5 min at 4°C. The supernatants were then collected and measured using the BCA protein assay kit (Thermo, USA). Protein samples (10–20 μg) were separated by 6–15% denatured polyacrylamide gels and transferred to PVDF membranes (Millipore, MA, USA) in transfer buffer with an ice‐water bath. After being blocked with 5% (w/v) nonfat milk for 1 h at room temperature, the membranes were incubated with specific primary antibodies (usually 1:1000 dilutions) overnight at 4°C. After being washed three times with Tris‐buffered saline with 0.1% Tween 20 (TBST), the membranes were probed with HRP‐linked secondary antibodies (usually 1:10,000 dilutions) for 1 h at room temperature. Thereafter, the immunoblotting was detected using an ECL detection reagent and scanned using a DS‐6500 scanner (EPSON, Japan). Finally, the signal intensity was quantified as integrated optical density using ImageJ software.

### 2.6 Myeloperoxidase (MPO), malondialdehyde (MDA), and nitric oxide (NO) assay

Colons were homogenized in tissue lysis buffer and then centrifuged at 4°C for 15 min at 12,000 *g*. The supernatant was collected and used for MPO, MDA, and NO analyses. The MPO activity was measured using commercial kits (JianCheng Bio, Nanjing, China) according to the manufacturer's instructions. The lipid peroxidation status was expressed as MDA concentration and determined by the thiobarbituric acid reactive substances method. The production of NO was assayed with Griess reagent by determining the amount of nitrite. Data on the activities of MPO were expressed as units per milligram of protein (U/mg protein), and the results of MDA and NO were expressed as nmol/mg protein.

### 2.7 Enzyme‐linked immunosorbent assay (ELISA)

The levels of inflammatory cytokines (TNF‐α, IL‐1β, and IL‐6) in colon tissues were measured by ELISA according to the manufacturer's protocols. Absorbance was measured using a SpectraMax® iD3 microplate reader (Molecular Devices, CA, USA) at 450 nm. And the results were extrapolated from the standard curve and expressed as pg/mL.

### 2.8 Immunohistochemistry

Immunohistochemistry was performed on 5 μm paraffin‐embedded sections of colon tissue. After deparaffinization and rehydration, 3% H_2_O_2_ was used to block the endogenous peroxidase activity, and then antigen retrieval was performed in sodium citrate buffer (pH 6.0) for 10 min by microwaving the sections. The slides were blocked with sheep serum for 15 min at room temperature. Then, the sections were immunostained with primary F4/80 antibodies (1:100) overnight at 4°C. Afterward, sections were incubated with secondary antibodies for 20 min at room temperature, counterstained with hematoxylin for 3 min, and rinsed in tap water for 5 min. Finally, sections were photographed under the fluorescence microscope (Olympus, Japan).

### 2.9 Statistical analysis

The data were expressed as the mean ± standard deviation (mean ± SD). SPSS version 16.0 (SPSS, Chicago, IL, USA) and GraphPad Prism 8 (La Jolla, CA, USA) were used for statistical analysis. Group means were compared using a one‐way analysis of variance (ANOVA) followed by Tukey's test. *p* < .05 was considered statistically significant.

## 3 RESULTS

### 3.1 DP alleviated the clinical symptoms of DSS‐induced colitis in mice

As shown in Figure 1b, at the end of the experiment, the body weight reduced significantly after DSS treatment, while the reduction was suppressed by DP pretreatment (*p* < .01). DSS treatment also resulted in a significant increase in DAI (*p* < .01), which was significantly reversed by DP treatment (*p* < .01). When the experiment was finished, the model group was 3.61 ± 0.15, while the DSS + DP (L) and DSS + DP (H) groups were 2.60 ± 0.16 and 1.82 ± 0.16, respectively. Colorectal shortening also reflected the extent of colon damage during acute DSS‐induced colitis (Tang et al., 2020). DSS‐treated mice showed shorter colons than the control group (6.05 ± 0.60 vs 9.58 ± 0.54 cm, *p* < .01), whereas the colon length in the DSS + DP(L) and DSS + DP(H) groups as well as the DP group (6.89 ± 0.49, 7.59 ± 0.75, 8.02 ± 0.48) had a dramatic increase compared with the DSS group (*p* < .01) (Figure 1d,e).

### 3.2 DP alleviated histological damage and inflammation responses in DSS‐induced colitis

**FIGURE 2:**
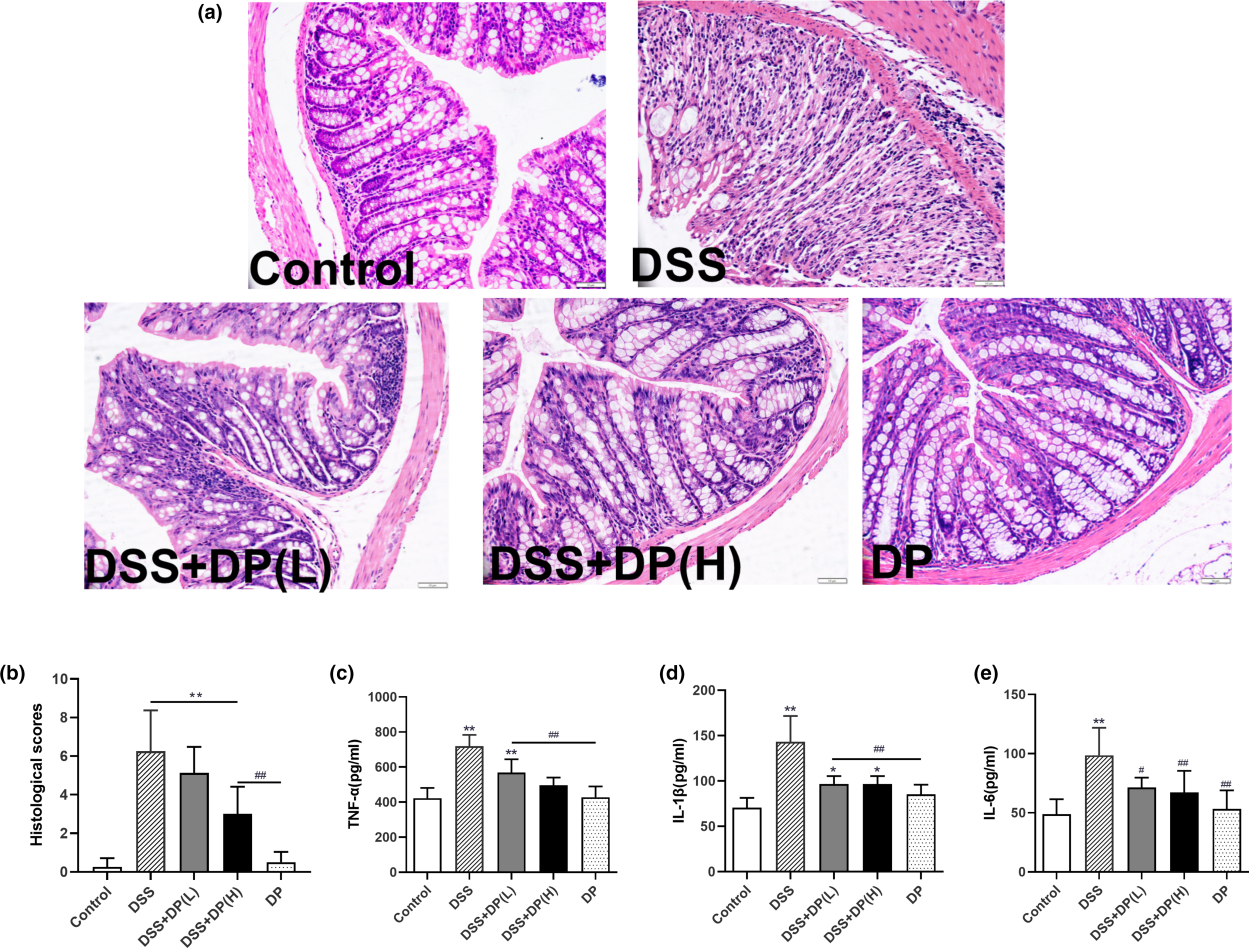
Effect of DP on the histopathological changes and proinflammatory cytokine expression of DSS‐induced ulcerative colitis in mice. (a) Histological changes in colonic tissue were determined using H&E staining (200×). (b) Histological scores of colon tissue. (c–e) Protein levels of TNF‐α, IL‐1β, and IL‐6 levels in colon tissue. Data are presented as the mean ± SD (*n* = 8), *p < .05, **p < .01 vs control group; ^#^ *p* < .05, ^##^ *p* < .01 vs DSS group.

To evaluate the protective effects of DP on DSS‐induced colitis, the histopathology of colon tissue was chosen to further analyze. The colonic epithelium consists of a variety of epithelial cells, such as enterocytes, goblet cells, Paneth cells, and enteroendocrine cells. As shown in Figure 2a,b, H&E staining showed that DSS induced a severe loss of epithelial integrity and serious disruption of crypt architecture and caused a high histological score. Treatment with DP alleviated the damage to colon tissues, improved the intestinal epithelial, decreased inflammatory cell infiltration, and the pathological scores were also significantly decreased (*p* < .05).

Another major central feature of DSS‐induced colitis in mice is the increased expression of proinflammatory cytokines. As depicted in Figure 2c–e, the levels of TNF‐α, IL‐1β, and IL‐6 in the DSS group were all significantly increased compared with the control group (*p* < .01). However, DP treatment downregulated the production of these proinflammatory cytokines (*p* < .05). The levels of TNF‐α, IL‐1β, and IL‐6 in the DSS + DP (H) group even reduced to the normal level and showed no significant difference compared with the control group (*p* > .05).

### 3.3 DP inhibited the infiltration of inflammation‐regulation cells in DSS‐induced colitis

**FIGURE 3:**
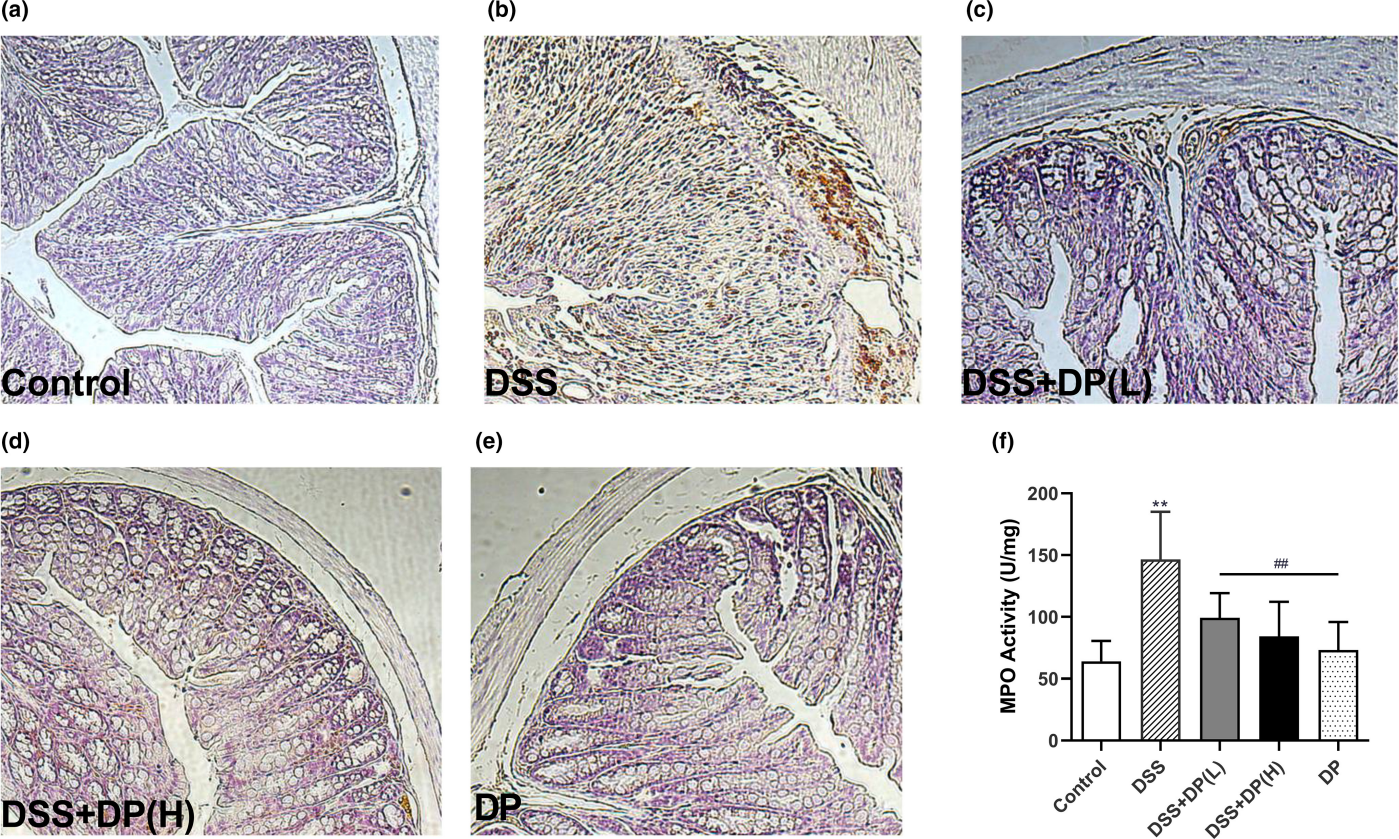
DP reduces the infiltration of macrophages and neutrophils. (a–e) The macrophages expressing F4/80 in colon tissue were monitored via immunohistochemical staining. Images of the colon tissues were obtained under a microscope at a magnification of ×200. (f) The MPO activity in colon tissues was determined by ultraviolet spectrophotometry. Data are presented as the mean ± SD (*n* = 8), *p < .05, **p < .01 vs control group; #*p* < .05, ##*p* < .01 vs DSS group.

The initial colon tissue damage caused by DSS induced a heavy influx of inflammatory cells, such as infiltrating neutrophils and macrophages. F4/80 antigen, as part of the EGF‐TM7 family, is expressed on a wide range of mature tissue macrophages (Li et al., 2020). The results of immunohistochemical staining shown in Figure 3a–e demonstrated that nearly no F4/80 positive expression was found in colon tissues of the control group and DP group, whereas DSS, DSS + DP(L), and DSS + DP(H) groups showed F4/80 positive expression with an inhomogenous degree. The highest expression level was found in the DSS group, followed by DSS + DP (L) and DSS + DP (H).

MPO activity, a parameter of neutrophil infiltration, has also been widely used to detect and monitor intestinal inflammation (Brito et al., 2016). Figure 3f depicts the MPO activity in colon tissue of the DSS‐treated group, which showed a marked increase of 128% compared to that in the control group (*p* < .01). In contrast, treatment with DP resulted in dramatically decreased MPO activity compared with the DSS group (*p* < .01). Thus, our results showed that DP had a protective effect on DSS‐induced colitis by decreasing the infiltration of inflammation‐regulating cells.

### 3.4 DP repressed NF‐κB activation in DSS‐induced mice colonic tissue

**FIGURE 4:**
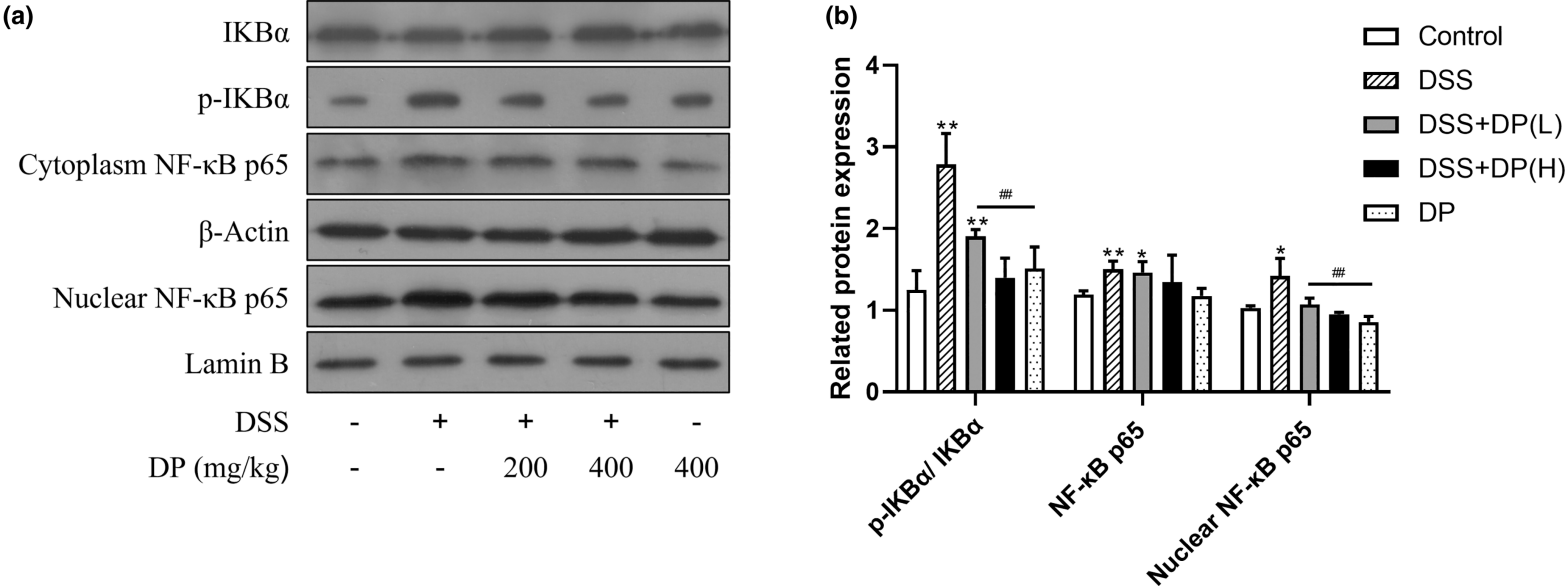
DP treatment downregulates the NF‐κB signaling pathway in DSS‐induced colitis. Representative blots (a) and quantitative data (b) of IKBα, p‐IκBα, and NF‐κB p65 in colonic tissue are shown. Data are presented as the mean ± SD from at least 3 independent experiments and expressed as the percentage of the control. *p < .05, **p < .01 vs control group; ^#^ *p* < .05, ^##^ *p* < .01 vs DSS group.

The nuclear factor kappa B (NF‐κB) is a critical transcriptional factor that regulates the expression of pro‐inflammatory cytokines in the intestinal inflammatory process (Atreya et al., 2008). In this study, the protein expression of cytoplasmic inhibitor of kappa B alpha (IKBα), phosphorylation of IκBα (p‐IKBα), NF‐κB p65, and nuclear NF‐κB p65 were examined by Western blotting. As shown in Figure 4, the expression of IκBα in the cytoplasm shows no significant difference between all five groups (*p* > .05). However, p‐IκBα and the ratio of p‐IκBα/IκBα were significantly increased in the DSS group compared with the control group (*p* < .01). After DP treatment, the ratio of p‐IκBα/IκBα was decreased in the DSS + DP (H) and DP groups compared with the DSS group (*p* < .01). Beyond that, the NF‐κB p65 and nuclear NF‐κB p65 protein expression were increased in the DSS group compared with the control group (*p* < .01), and the DP treatment led to a significantly decreased expression compared with the DSS group (*p* < .05).

### 3.5 DP inhibits protein expression of the NLRP3 inflammasome complex and downstream inflammatory cytokines in DSS‐induced colitis

**FIGURE 5:**
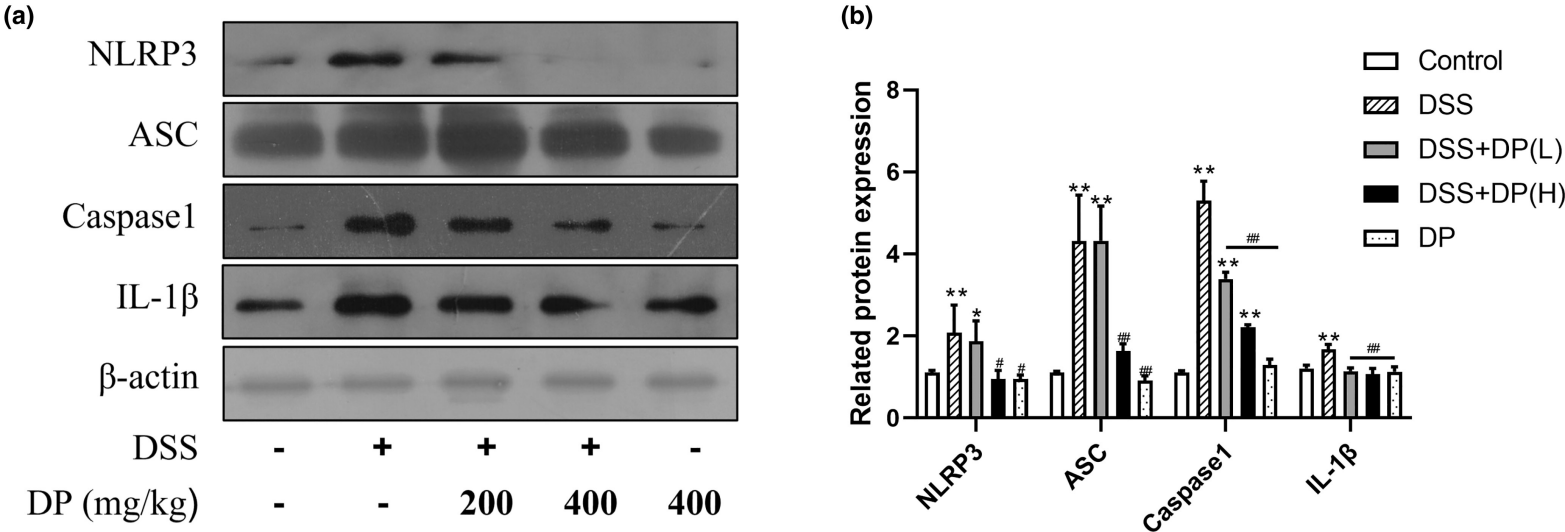
DP inhibits DSS‐induced activation of the NLRP3 inflammasome in the colon tissues of mice. (a) Expression of NLRP3, ASC, caspase‐1, and IL‐1β were detected by Western blot, and the representative blots are shown. (b) The density of NLRP3, ASC, caspase‐1, and IL‐1β was quantified. Data are presented as the mean ± SD from at least 3 independent experiments and expressed as the percentage of the control. *p < .05, **p < .01 vs control group; ^#^ *p* < .05, ^##^ *p* < .01 vs DSS group.

The NLRP3 inflammasome has been recognized as a potential therapeutic target of intestinal inflammation in the DSS colitis model (Mei et al., 2019). The effect of DP treatment on NLRP3 inflammation‐related signal pathways was examined by Western blotting (Figure 5). The results showed that the expression levels of NLRP3, ASC, IL‐1β, and Caspase‐1 in the DSS group were significantly increased by 108%, 331%, 430%, and 41%, respectively, when compared with the control group (*p* < .01), which were markedly decreased with DP pretreatment when compared with the DSS group (*p* < .05), except for Caspase‐1 in the DSS + DP (L) group (*p* > .05). These results indicated that DP can inhibit the expression of the NLRP3/ASC/Caspase‐1/ IL‐1β pathways in DSS‐induced colitis.

### 3.6 DP treatment abrogated oxidative stress and prevented the suppression of the Nrf2 pathway in DSS‐induced colitis

**FIGURE 6:**
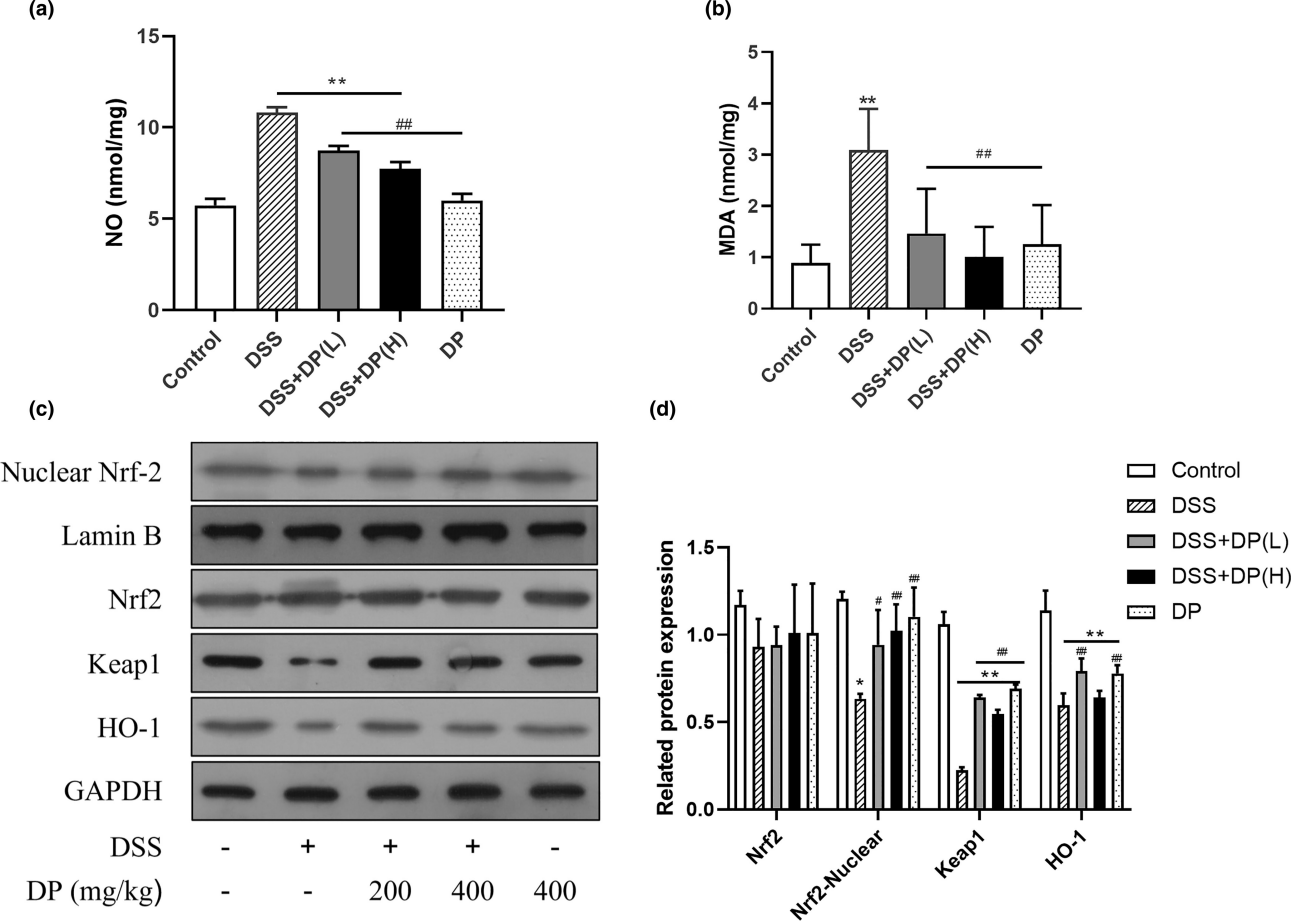
DP prevented the DSS‐induced oxidative stress with decreased levels of NO, MDA, and suppression of the Nrf‐2 pathway. NO level (a), MDA level (b), representative blots (c), and the qualitative data (d) of Nrf2, Keap1, and HO‐1 in DSS‐induced colonic tissue are shown. Data are presented as the mean ± SD from at least 3 independent experiments and expressed as the percentage of the control. *p < .05, **p < .01 vs control group; ^#^ *p* < .05, ^##^ *p* < .01 vs DSS group.

NO and MDA are two typical indexes that play a significant role in oxidative stress. To investigate the effect of DP on oxidative stress, the levels of NO and MDA were detected in DSS‐induced colitis. As shown in Figure 6a,b, compared with the control group, the levels of NO and MDA increased significantly in the DSS group (*p* < .01). But these levels were remarkably inhibited after DP treatment in a dose‐dependent manner, indicating that DP treatment significantly suppressed the DSS‐induced increase of MDA and NO levels.

Nuclear factor erythroid 2‐related factor 2 (Nrf2) is a transcription factor that plays a key role in the activation of cellular antioxidant enzymes in response to oxidative stress (Zhao et al., 2019). It has been reported that Nrf‐2 signaling was identified as a critical mechanism for chemical‐induced UC (Serrya et al., 2021). To investigate whether Nrf‐2 signaling regulated DSS‐induced colitis, we detected the protein levels of Nrf‐2, Keap‐1, and HO‐1 by Western blot. As shown in Figure 6c,d, we found that the total Nrf2 protein level had no significant difference between the groups. While the nuclear Nrf2 protein level in the DSS group decreased obviously (*p* < .05), so did the decline of Nrf2‐regulated Keap‐1 (*p* < .01) and HO‐1 (*p* < .01) protein levels compared with the control group. As expected, DP pretreatment inhibited the decrease of these antioxidant enzymes, especially Nrf‐2 (*p* < .05) and Keap‐1 (*p* < .01), compared with the DSS group.

## 4 DISCUSSION

Due to its pathological similarity to human UC, DSS‐induced colitis is widely accepted and used to explore and evaluate the efficiency of protective agents (Eichele & Kharbanda, 2017). A large number of studies have demonstrated the therapeutic efficacy of DP for various disease models (Cai et al., 2017; Ren et al., 2019), but no study has shown the effect of DP on colonic inflammation. In the present study, we aimed to evaluate the effect of DP on DSS‐induced UC and elucidate the potential molecular mechanisms underlying these protective effects in mice.

Previous studies have demonstrated that the derangement of cytokines drives colitis pathology and disease progression (Ranson et al., 2018; Waldner & Neurath, 2014), TNF‐α, IL‐1β, and IL‐6 are important pro‐inflammatory effectors that function in the context of intestinal inflammation. They could activate the target immune cells and further aggravate inflammation. Blockade of these pro‐inflammatory cytokines signaling with monoclonal antibodies was effective in suppressing colitis. In the current study, we found that the protein levels of TNF‐α, IL‐1β, and IL‐6 were all significantly increased after DSS exposure, which were closely correlated with disease severity. Furthermore, we also found that DP treatment significantly reduced the production of pro‐inflammatory cytokines in the colonic tissue, accompanied by the alleviation of colitis pathological manifestations, which implied that the suppression of pro‐inflammatory cytokines might contribute to the protective effect of DP against DSS‐induced colitis. Along with pro‐inflammatory cytokine secretion, recruitment and infiltration of innate immune system cells, especially neutrophils and macrophages, are also recognized as evaluative parameters for the severity and degree of colitis (Yan et al., 2018). Although the contribution of neutrophils to the pathogenesis of UC remains controversial, their infiltration extent correlates with the severity of the disease and is included in the scoring system for UC severity (Bressenot et al., 2015). The state of macrophage polarization is also critical in defining the resolution or progression of inflammation and disease (Moreira Lopes et al., 2020). The current study revealed that DSS exposure causes significant infiltration and activation of neutrophils and macrophages, which is completely abrogated by DP treatment.

NF‐κB has been recognized as a critical transcriptional factor that regulates the expression of pro‐inflammatory cytokines in the intestinal inflammatory process (Atreya et al., 2008). In the resting state, NF‐κB exists in the cytoplasm as an inactive form. In response to a variety of stimuli, such as oxidants and cytokines (e.g. TNF‐α and IL‐1β), the activated IκB kinase (IKK) complex mediates the phosphorylation of IκB and causes the release and nuclear translocation of NF‐κB dimers, the majority of which are composed of p50 and p65 (RelA). The nuclear translocation of NF‐κB activates its target genes to promote the expression of various pro‐inflammatory cytokines and mediators. Furthermore, NF‐κB‐induced inflammatory cytokines, in turn, potentiated the activation of NF‐κB, and this positive feedback contributes to the constitutive activation of colitis inflammation (Du et al., 2017). Previous studies have demonstrated that the expression and activation of NF‐κB are strongly induced in the inflamed gut of IBD, and NF‐κB suppression by a pharmacological inhibitor or genetic manipulation could protect against experimental colitis in mice (Hirata et al., 2007). Therefore, blocking the activation of NF‐κB may be an effective strategy for UC management. In this regard, our study revealed that the remarkably increased phosphorylation level of IκB and upregulation of nuclear NF‐κB p65 induced by DSS in mice colonic tissues was partly suppressed by DP. In accordance with this, previous studies have found that DP could inhibit NF‐κB‐mediated inflammation in liver tissue and in LPS‐induced RAW264.7 cells (Park et al., 2010, 2014). These results suggest that suppressed NF‐κB transactivation might contribute to the protective effects of DP against the experimental UC model.

Some cytokines, such as IL‐1β and IL‐18, whose pro‐forms are primed by NF‐κB transactivation, require subsequent proteolytic cleavage for maturation. This second step is performed by caspase‐1 in the inflammasome. The NLRP3 inflammasome is the most extensively characterized inflammasome and has been identified as a critical mechanism of intestinal inflammation (Bauer et al., 2010). The multiprotein complex of NLRP3 inflammasome consists of the pattern recognition receptor NLRP3, the adaptor apoptosis‐associated speck‐like protein containing a CARD (ASC), and the effector caspase‐1. When NLRP3 identifies stimuli, it subsequently polymerizes and recruits pro‐caspase‐1 through ASC, and then the inflammasome cleaves pro‐caspase‐1 into caspase 1. The active caspase‐1 further converts pro‐IL‐1β and pro‐IL‐18 into their active forms‐IL‐1β and IL‐18. In addition, caspase‐1 is also involved in a form of inflammatory cell death termed pyroptosis. For the pathogenesis of colitis, the detailed roles of the NLRP3 inflammasome are controversial. Several studies have revealed that mice with genetically deficient NLRP3 or suppressed by an inhibitor experience less severe pathology (Bauer et al., 2010). In contrast, some researchers suggested that the NLRP3 inflammasome activation exhibits a protective role in intestinal inflammation (Itani et al., 2016). The different results might be attributed to the variations in genetic backgrounds, gut microbiota, colitis models, or approaches to induce colitis, which need further research (Zhen & Zhang, 2019). In the present study, the administration of DP suppressed the activation of NLRP3 and downregulated the expression of IL‐1β induced by DSS. These results indicated that the alleviative effects of DO on colitis might relate to the inhibition of the NLRP3 inflammasome.

The present study also investigated the involvement of oxidative stress and the Nrf2 signaling pathway in the protection of DP against DSS‐induced UC. Increased ROS and decreased antioxidant activity contribute to the pathogenesis of IBD (Bhattacharyya et al., 2014). Excessive levels of ROS increase epithelial barrier permeability and pathogen invasion, exaggerate inflammatory cell infiltration, and cause inflammatory damage (Wang et al., 2016). The Nrf2‐regulated antioxidant system is an important scavenger system that protects cells against oxidative stress, and Nrf2 has been implicated as a promising therapeutic target for the treatment of various gastrointestinal disorders (Yanaka, 2018). Once dissociated with Keap1 in the cytoplasm, Nrf2 translocates into the nucleus, binds to antioxidant response elements, and then leads to the overexpression of antioxidant enzymes such as HO‐1 and SOD. In this study, DSS treatment caused a significant increase in oxidative stress and downregulation of the Nrf2 signal pathway. Interestingly, DP significantly suppressed the DSS‐induced increase in MDA and NO levels, together with the increased expression of Nrf2 and its targeted genes. Previously, studies have demonstrated that DP are novel nature antioxidants and could upregulate HO‐1 expression through the Nrf2 signaling pathway in vitro (Park et al., 2014). Therefore, we speculated that inhibition of oxidative stress and activation of the Nrf2/HO‐1 pathway are involved in the beneficial effects of DP in the DSS‐induced UC mice model.

Oxidative stress and inflammation play important roles in the pathogenesis of UC and correlate with each other. Excessive ROS are produced, leading to oxidative stress during the inflammatory response, especially by the neutrophils during phagocytosis. In return, ROS could function as second messengers to stimulate nuclear translocation of NF‐κB and activation of the NLRP3 inflammasome to exaggerate inflammation (Tschopp & Schroder, 2010). On the other hand, Nrf2, as a central transcriptional factor in the cellular antioxidative system, also crosstalks with the inflammatory pathways. Nrf2 and NF‐κB signaling pathways mutually inhibit the transcription or function of each other's downstream target proteins (Ahmed et al., 2017). Furthermore, Nrf2 nuclear translocation could also inhibit the activation of the NLRP3 inflammasome (Liu et al., 2016). Therefore, activation or inhibition of one pathway might be a result of the other during DP treatment of UC, which needs further research. The elucidation of the mechanisms underlying this crosstalk is of great scientific interest and will also contribute to the treatment of colitis.

## 5 CONCLUSIONS

**FIGURE 7:**
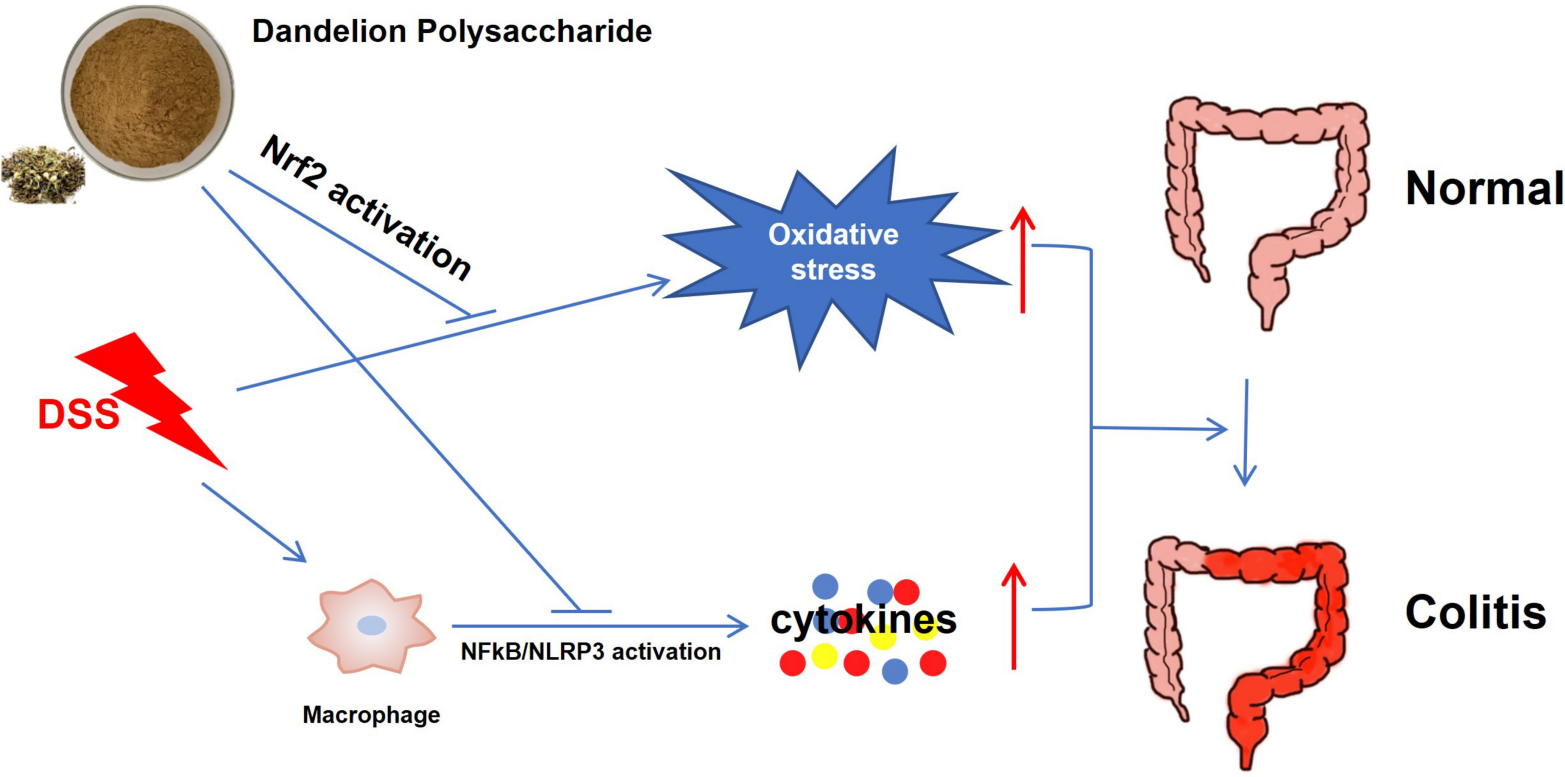
The possible mechanism for DP alleviation of DSS‐induced colitis in mice.

In conclusion, the current study indicated that oral administration of DP could alleviate the severity of DSS‐induced colitis, and the protection mechanisms might be related to its antioxidant and anti‐inflammation ability via the inhibition of NF‐κB signaling and the NLRP3 inflammasome, as well as the activation of the Nrf2 pathway (Figure 7). This study proposes that DP may be a promising pharmaceutical candidate for the treatment of UC.

## AUTHOR CONTRIBUTIONS

**Shuo Wang:** Conceptualization (lead); formal analysis (lead); investigation (equal); methodology (equal); writing – original draft (equal). **Ping Wu:** Investigation (equal); validation (lead); visualization (equal); writing – original draft (equal). **Zongqiang Fan:** Data curation (lead); software (equal). **Xingrui He:** Methodology (equal); resources (lead). **Jinqian Liu:** Software (equal); visualization (equal). **Ming Li:** Funding acquisition (equal); writing – review and editing (lead). **Fang Chen:** Funding acquisition (equal); project administration (lead); supervision (lead).

## FUNDING INFORMATION

This work was supported by the Natural Science Foundation of Shandong Province (ZR2019BB046, ZR2020MC126), the Open Project of Shandong Collaborative Innovation Center for Antibody Drugs (No. CIC‐AD1816), the Open Project of Liaocheng University Animal Husbandry Discipline (NO. 319312101‐28), and the Doctoral Foundation of Liaocheng University (318051744).

## CONFLICT OF INTEREST STATEMENT

The authors declare that the research was conducted in the absence of any commercial or financial relationships that could be construed as a potential conflict of interest.

## ETHICS STATEMENT

All animals' experiments were approved strictly by the Animal Care and Research Ethics Committee of Liaocheng University (Permit Number: 20200926) in accordance with the NIH Guide for Care and Use of Laboratory Animals.

## Supporting information


Table S1

## Data Availability

The original contributions presented in this study are included in the article; further inquiries can be directed to the corresponding authors.
