# Supplementary material for: Dandelion polysaccharide treatment protects against dextran sodium sulfate‐induced colitis by suppressing NF‐κB/NLRP3 inflammasome‐mediated inflammation and activating Nrf2 in mouse colon
Source: Food Sci Nutr. 2023 Sep 19;11(11):7271–82. doi: 10.1002/fsn3.3653 (PMC10630811; doi:10.1002/fsn3.3653)
Supplement: Supplementary file 1 — Table S1 [file FSN3-11-7271-s001.docx]

**Supplementary Table 1 Primary antibodies used in the study**

| Target protein | Manufacture | Catalog number |
| --- | --- | --- |
| IKBα (L35A5) | Cell Signaling Technology | 4814 |
| p-IKBα (Ser^32^)(14D4) | Cell Signaling Technology | 2859 |
| NF-κB p65 (D14E12) | Cell Signaling Technology | 8242 |
| HO-1 (E3F4S) | Cell Signaling Technology | 43966 |
| Nrf2 (D1Z9C) | Cell Signaling Technology | 12721 |
| NLRP3 (D2P5E) | Cell Signaling Technology | 13158 |
| IL-1β (3A6) | Cell Signaling Technology | 12242 |
| GADPH (1E6D9) | Proteintech | 60004-1-Ig |
| Caspase-1 | Proteintech | 22915-1-AP |
| Beta Actin (4H1) | Proteintech | 81115-1-RR |
| Lamin B | Proteintech | 12987-1-AP |
| ASC | Proteintech | 10500-1-AP |
| F4/80 (C-7) | Santa Cruz | sc-377009 |
